# A Novel GABA-Producing *Levilactobacillus brevis* Strain Isolated from Organic Tomato as a Promising Probiotic

**DOI:** 10.3390/biom15070979

**Published:** 2025-07-08

**Authors:** Asia Pizzi, Carola Parolin, Davide Gottardi, Arianna Ricci, Giuseppina Paola Parpinello, Rosalba Lanciotti, Francesca Patrignani, Beatrice Vitali

**Affiliations:** 1 Department of Pharmacy and Biotechnology, University of Bologna, 40127 Bologna, Italy; asia.pizzi2@unibo.it (A.P.); b.vitali@unibo.it (B.V.); 2 Department of Agricultural and Food Sciences, University of Bologna, 47521 Cesena, Italy; davide.gottardi2@unibo.it (D.G.); arianna.ricci4@unibo.it (A.R.); giusi.parpinello@unibo.it (G.P.P.); rosalba.lanciotti@unibo.it (R.L.); francesca.patrignani@unibo.it (F.P.)

**Keywords:** *Levilactobacillus brevis*, cell-free supernatants, gamma aminobutyric acid (GABA), probiotic, antimicrobial activity, anti-inflammatory activity

## Abstract

Gamma-aminobutyric acid (GABA) is a non-protein amino acid playing a significant role in the central nervous system and the gut–brain axis. This study investigated the potential to produce GABA by lactic acid bacteria (LAB) isolated from different varieties of organic tomatoes. The isolated LAB were taxonomically identified by 16S rRNA gene sequencing, the presence of the *gadB* gene (glutamate decarboxylase) was detected, and GABA production was quantified using HPLC. *Levilactobacillus brevis* CRAI showed the highest GABA production under optimised fermentation conditions with 4% monosodium glutamate (MSG). The genome sequencing of *L. brevis* CRAI revealed the presence of *gadA* and *gadB* isoforms and assessed the strain’s safety profile. The gene expression analysis revealed that the *gadA* and *gadB* genes were upregulated in the presence of 4% MSG. The probiotic potential of *L. brevis* CRAI was also assessed by functional assays. The strain showed strong antimicrobial activity against representative enteropathogens, i.e., *Escherichia coli* ETEC, *Salmonella choleraesuis*, and *Yersinia enterocolitica*, and anti-inflammatory effect, reducing nitric oxide production in LPS-stimulated RAW264.7 macrophages. In addition, its ability to adhere to intestinal epithelial Caco-2 cells was demonstrated. These results highlight *L. brevis* CRAI as a promising candidate for the development of GABA-enriched functional foods or probiotic supplements with the perspective to modulate the gut-brain axis.

## 1. Introduction

Gamma-aminobutyric acid (GABA) is a non-protein amino acid widely distributed in nature among microorganisms, plants, and animals [1]. GABA is a bioactive compound with multiple biological functions; indeed, it is one of the major inhibitory neurotransmitters responsible for transmitting chemical signals within the mammalian central nervous system [2]. Furthermore, GABA exhibits numerous physiological and pharmacological effects on different patho-physiological processes, such as brain development, stress regulation, blood pressure modulation, diuresis, and diabetes management [3,4]. It also plays a role in the gut–brain signalling through different pathways involving enteric neurons, entero-endocrine cells, and immune cells [5], although the precise role and functional importance of GABA within the enteric nervous system remain unclear. GABA potentially modulates the inhibition or stimulation of the gastrointestinal (GI) tract motor and secretory function by activating GABA receptors on the GABAergic neuronal cells along the GI tract [6]. Recent studies have proposed GABA as a potential key player in nerve-driven immunity, since several immune cells possess components of the GABAergic system, including GABA receptors and transporters [7]. GABA contributes to the immune response modulation by negatively regulating pro-inflammatory cytokine production and immune cell proliferation. Consequently, the GABAergic system fulfils the criteria to be recognised as a “connecting bridge” that facilitates the functional cooperation between the nervous and immune systems [8]. Due to its widespread physiological and pharmaceutical effects on humans, several studies have focused on the development of new and effective GABA-based therapeutic strategies for the treatment of different diseases, including those affecting the brain, nervous system, and cardiovascular system [9]. The biosynthesis of GABA occurs in one single step: the glutamic acid decarboxylase (GAD), a pyridoxal 5′-phosphate-dependent enzyme, catalyses the irreversible α-decarboxylation of L-glutamate into GABA [10]. Yeast, fungi, plants, and bacteria have been extensively studied as natural GABA producers [11]; in particular, the GAD enzyme is highly conserved within lactic acid bacteria (LAB) [12]. The so-called glutamic acid decarboxylase system enables LAB to withstand acid environments, such as the gastrointestinal tract, while maintaining viability and cellular activity [13]. This mechanism provides protection under acid conditions since decarboxylation of glutamate to GABA by the GAD enzyme consumes intracellular protons, thereby maintaining cytosolic pH homeostasis [14]. The glutamic acid decarboxylase system consists of the glutamate/GABA antiporter, encoded by the *gadC* gene, and the GAD enzyme, encoded by the *gadA* or *gadB* genes [15]. Specifically, the synthesis of GABA follows these steps: glutamate is transported into the cell through the antiporter, where it undergoes decarboxylation, and then the resulting GABA is exported outside the cell through the same antiporter [16]. In LAB, the *gad* operon is located on the chromosome, and its organisation varies among different species and strains [17]. LAB represents an important, widespread, and ubiquitous group of Gram-positive bacteria, characterised by unique physiological activities that have been extensively exploited in the food industry [18,19,20]. Additionally, many LAB exhibit health-promoting properties that make them potential probiotics [21]. Among LAB, most of the GABA-producing strains belong to *Lactobacillus* and related genera [22], which have traditionally been applied in the dairy industry and food supplements. Lactobacilli are facultative anaerobic, acid-tolerant, non-spore-forming rods that often grow under microaerophilic conditions. Several studies have demonstrated the therapeutic effects of beneficial lactobacilli [23,24], which are widely used as probiotics [25].

In this study, a collection of LAB strains, belonging to *Levilactobacillus brevis*, *Lactiplantibacillus plantarum*, and *Lactococcus lactis* species, was isolated from different varieties of organic tomatoes and evaluated for their ability to produce GABA. We selected a *L. brevis* strain, namely *L. brevis* CRAI, which exhibited the best GABA production performance under optimised fermentation conditions [26]. Additionally, we analysed its genome sequence and characterised some functional properties, aiming to explore its potential as a novel probiotic.

## 2. Materials and Methods

### 2.1. Isolation of Lactic Acid Bacteria from Tomato Source

Lactic acid bacteria (LAB) were isolated from different varieties of organic tomatoes picked in the Bologna surrounding countryside. Tomato pulp was blended in De Man, Rogosa, and Sharp (MRS) broth (Difco, Detroit, MI, USA) supplemented with 0.05% L-cysteine (Merck, Milan, Italy). Different concentrations of the mixture were cultured on MRS plates and incubated for 72 h at 37 °C in anaerobic jars containing GasPak™ (GasPak™ EZ Anaerobe Container System, Sparks, MD, USA). Colonies with different morphologies yielding variable rods by microscope observation were selected for glycerol stock preparation. Isolates were routinely grown in MRS medium supplemented with 0.05% L-cysteine, at 37 °C, in anaerobiosis.

### 2.2. Taxonomic Identification of the Isolates by 16S rRNA Gene Sequence

Isolates were cultured overnight, then 2 mL were pelleted (10 min at 10,000× *g*) and genomic DNA was extracted by using the DNeasy Blood & Tissue Kit (Qiagen, Hilden, Germany) following the protocol “Pretreatment for Gram-positive bacteria” [26]. The complete 16S ribosomal RNA (rRNA) gene was amplified with the universal primers 27F and 1492R [27] and sequenced. The obtained sequences were compared with the sequences available in BLASTn (https://blast.ncbi.nlm.nih.gov, accessed on 15 January 2024) to reach the taxonomic identification of the isolates.

### 2.3. Molecular Detection of gadB Gene

The genomic DNA samples obtained from LAB isolated from tomatoes were used to check for the presence of the *gadB* gene, encoding for glutamate decarboxylase. The PCR primers, designed on highly conserved regions of the *gadB* sequence, were as follows: CoreF 5′-CCTCGAGAAGCCGATCGCTTAGTTCG-3′, CoreR 5′-TCATATTGACCGGTATAAGTGATGCCC-3′ for *L. brevis* and *L. plantarum* strains [28], and Forward 5′-CAACATGATCGCTGACCTTTGG-3′ and Reverse 5′-GCCATTCCACCAAGCATACAAG-3′ for *L. lactis* strains [29].

### 2.4. Quantitative Analysis of GABA Production by High-Performance Liquid Chromatography (HPLC)

LAB strains positive for the *gadB* gene were grown in the optimal fermentation conditions to obtain maximum GABA yield, following the parameters reported in the literature [18,22,30,31,32]. Specifically, the strains were grown in MRS broth supplemented with 0.05% L-cysteine at 32 °C for 48 h under anaerobic conditions; the medium was supplemented with different concentration of L-glutamic acid monosodium salt (MSG, ThermoFisher, Kandel, Germany) from 0 mM (0%) to 236.53 mM (4%). Then, the initial pH was adjusted to 5, and the initial inoculum was 10^7^ CFU/mL. After 48 h of incubation, culture supernatants were collected by centrifugation at 5000× *g* for 10 min at 4 °C and filtered with 0.22 µm membrane filters, and then used for GABA quantification. GABA was derivatised through the combination of 1.75 mL of 1 M borate buffer (pH 9), 750 µL of methanol, 1 mL of the sample, and 20 µL of diethyl ethoxymethylenemalonate (DEEMM). The resulting mixture was subjected to ultrasonic treatment for a duration of 30 min [33]. The GABA derivative was analysed and quantified by Agilent Infinity 1290 HPLC-DAD (Agilent Technologies, Waldbronn, Germany) using the binary gradient reported by Gòmez-Alonso et al., 2007 (phase A, 25 mM acetate buffer pH 5.8 with 0.02% sodium azide; phase B, 80:20 mixture of acetonitrile and methanol; flow rate 0.9 mL/min) [34]. As a minor modification, the injection volume was set to 1 µL, and the chromatography run was shortened to 40 min compared to the original 80 min, using an isocratic eluent A (5 min) to rebalance the column in between samples. The area of the peak corresponding to GABA was used for quantification by comparing it with a standard curve, which was generated by derivatising commercial GABA from Merck (Merck Millipore, Darmstadt, Germany) and analysing it under the same conditions. The GABA concentration used for the calibration curve ranged from 0.96 to 29.09 mM (R^2^ = 0.999).

### 2.5. Genome Sequencing of Levilactobacillus brevis CRAI and Analysis

*L. brevis* CRAI DNA extracted as described above was quantified by Qubit dsDNA BR assay kit using the Qubit 2.0 fluorometer (Life Technologies, Monza, Italy) and then sequenced by Oxford Nanopore Whole Genome Sequencing technology. Briefly, the genomic library was prepared using the SQK-LSK109 and EXP-NBD196 kits (Oxford Nanopore Technologies, Oxford, UK) and sequenced in an R9.4.1 flow cell (MIN106D, Oxford Nanopore Technologies, Oxford, UK) with the GridION X5 Nanopore device (Oxford Nanopore Technology, Oxford, UK). The genome was assembled de novo using Canu assembler v2.2 [35] and annotated by Bakta v1.4.1 [36] with a genome coverage of 39.47. The *L. brevis* CRAI genome was searched for the presence of antibiotic resistance and virulence-related genes using three different tools, i.e., AMR Finder Plus software (https://www.ncbi.nlm.nih.gov/pathogens/antimicrobial-resistance/AMRFinder/, version 4.0.15, updated on 15 September 2024), the ResFinder 4.6.0 database (https://cge.food.dtu.dk/services/ResFinder/ accessed on 15 September 2024, threshold for identity: 90.0%, minimum length: 60.0%), and Resistance Gene Identifier (RGI 6.0.3) using the Comprehensive Antibiotic Resistance Database (CARD, version 3.3.0; https://card.mcmaster.ca/ accessed on 15 September 2024). The aminoacidic sequence of GAD system proteins from *L. brevis* ATTC367, *L. brevis* CRL2013, *L. brevis* NCL912, *L. brevis* CD0817, *L. brevis* CRL2013, and *L. brevis* YSJ3 were retrieved from the NCBI database and aligned on BLASTp (https://blast.ncbi.nlm.nih.gov accessed on 15 June 2025) to calculate similarity scores.

### 2.6. gadA and gadB Gene Expression Analysis by RT PCR

*L. brevis* CRAI was cultured in optimised conditions as described in Section 2.4. with the addition of 0% and 4% of MSG in the medium. After 48 h of culture, total RNA was extracted using the RNeasy kit following the protocol “Enzymatic Lysis, Proteinase K Digestion and Mechanical Disruption of Bacteria” (Qiagen, Hilden, Germany) [37]. A total of 1 µg of extracted RNA was subjected to reverse transcription using the QuantiTect^®^ Reverse Transcription kit (Qiagen, Hilden, Germany); cDNA was used in quantitative Real-Time PCR (RT PCR) with a LightCycler 2.0 instrument (Roche, Mannheim, Germany) and LightCycler^®^ FastStart DNA Master SYBR Green I (Roche, Mannheim, Germany). At the end of each reaction, amplicon specificity was verified by first-derivative melting curve analysis, and the integrity of the PCR product was assessed by agarose gel electrophoresis. Relative expression of the *gadA* gene (5′-CAGGTTACAAGACGATCATGC-3′, 5′-ATACTTAGCCAGCTCGGACTC-3′ primers [38]) and *gadB* gene (Core F and Core R primers) was normalised to the 16S rRNA gene (HDA1 5′-ACTCCTACGGGAGGAGGCAGCAGT-3′, HAD2 5′-GTATTACCGCGGCTGCTGGCAC-3′ primers [39]) considering crossing point (CT) values. The fold of expression of the *gadA* and *gadB* genes was calculated following the 2^−∆∆CT^ method [40].

### 2.7. Assessment of Antimicrobial Activity of L. brevis CRAI

Cell-free supernatant (CFS) of *L. brevis* CRAI was collected by centrifugation of an overnight culture at 5000× *g* for 10 min at 4 °C and filtered through a 0.22 mm membrane. The pH value was measured by a pH meter. The antimicrobial activity of CFS was assayed against the following entero-pathogens: *Escherichia coli* ETEC H10407; *Salmonella choleraesuis* serovar Typhimurium, cultured twice in Nutrient Broth (NB) (Difco, Detroit, MI, USA); and *Yersinia enterocolitica*, cultured twice in Tripticase Soy Broth (TSB) (Difco, Detroit, MI) before each experiment. The experiment was performed in flat-bottom 96-well plates filled with 100 µL of *L. brevis* CFS and subsequently inoculated with 100 µL of the pathogen suspension (1 × 10^6^ CFU/mL). A growth control well contained 100 µL of sterile MRS medium and 100 µL of the same pathogens’ suspension. The plates were incubated at 37 °C for 24 h. Pathogen growth was analysed by reading the absorbance at 600 nm (EnSpire Multimode Plate Reader, PerkinElmer Inc., Waltham, MA, USA). Residual growth in the presence of *L. brevis* CFS was calculated as a percentage relative to the absorbance of the corresponding control. Each experiment was repeated at least six times, in two independent runs.

To evaluate the ability of *L. brevis* CRAI cells to antagonise the growth of intestinal pathogens, the protocol reported by Siroli et al. (2017) [41] was used. The pathogens used were previously mentioned. Briefly, 5 µL of the overnight cultures of *L. brevis* CRAI (approximately 5 × 10^8^ CFU/mL) was spotted over the surface of MRS plates and allowed to grow under anaerobic conditions at 37 °C for 24 h. Then, 0.1 mL (corresponding to 10^8^ CFU/mL) of an overnight culture of pathogens was inoculated into 10 mL of NB (*E. coli* ETEC H10407, *S. choleraesuis* serovar Typhimurium) or TSB (*Y. enterocolitica*) soft agar (containing 0.7% agar) and poured on the plates where *L. brevis* CRAI had grown. After 24 h at 37 °C, the plates were checked to evaluate the growth inhibition zones. The inhibition halos were measured from the outer perimeter of the spots in four directions, and the average radiant was considered. According to the radius of inhibition, the antagonistic activity showed by the strain was expressed as +, radius between 14–18 mm; ++, radius > 18 mm.

### 2.8. Inhibition of NO Production in LPS-Stimulated RAW 264.7 Cells

A murine macrophage cell line (RAW264.7) was grown in Dulbecco’s minimal essential medium (DMEM) (EuroClone, Pero, Italy), supplemented with 10% foetal bovine serum (Gibco Life Technologies Corporation, Segrate, MI, Italy), 1% L-glutamine (Merck, Saint Louis, MO, USA), and incubated in 5% CO_2_ atmosphere at 37 °C. Nitrite presence was measured in cell culture media by using the Griess Reagent Nitrite Measurement Kit (Cell Signalling technology, Inc., Trask Lane, Danvers, MA, USA) [42]. The Griess assay offers an indirect method for detecting Nitrite Oxide (NO) by quantifying nitrite, nitrate, and nitrosating agents. RAW264.7 cells were seeded into 24-well plates with a density of 1 × 10^5^ cells/wells and cultured for 48 h in complete medium. The inflammation process was activated by cell stimulation with 1 µg/mL bacterial LPS (LPS from *E. coli*, Enzo Life Sciences, Serotype TLRGRADE), and RAW264.7 was also treated with *L. brevis* CRAI bacterial cells or CFS at a ratio of 1:100 (eucaryotic cell: bacteria) for 24 h. After the treatment period, 100 µL of cell culture media was removed and added to 100 µL of Griess reagent in a 96-well plate, followed by spectrophotometric measurement at 550 nm (EnSpire Multimode Plate Reader, PerkinElmer Inc., Waltham, MA, USA). The inflammation was calculated as a percentage relative to the absorbance of the control, in which RAW 264.7 cells were stimulated by LPS alone (considered as 100% of inflammation). Each experiment was repeated at least three times, in two independent runs.

### 2.9. Adhesion of L. brevis CRAI to Caco-2 Cells

The colonocyte-like cell line Caco-2 was used to determine the adhesion ability of *L. brevis* CRAI strain following the method previously reported in [43]. Caco-2 cells were grown on glass coverslips, seeded at a concentration of 5 × 10^4^ cells/mL in Dulbecco’s minimal essential medium (DMEM) (EuroClone, Pero, Italy), supplemented with 10% foetal bovine serum, 1% L-glutamine, and incubated in 5% CO_2_ atmosphere at 37 °C. After 48 h (about 70% confluent), Caco-2 cells were washed twice in PBS, and then the complete medium was added. An overnight culture of *L. brevis* CRAI was washed twice in PBS and resuspended in DMEM at a concentration of 10^8^ CFU/mL. Caco-2 cells were incubated with *L. brevis* CRAI suspension at a ratio of 1:100 (eucaryotic cell: bacteria) for 3 h at 37 °C in 5% CO_2_. Afterwards, cell monolayers were washed several times in PBS, fixed with May-Grünwald, and stained with Giemsa. Results were read at light-microscopy (1000×) by counting the number of *L. brevis* bacterial cells attached to 200 randomly chosen Caco-2 cells.

### 2.10. Nucleotide Sequence Accession Number and Strain Deposit

The nucleotide sequences of the 16S rRNA genes of the isolated strains have been deposited in the GeneBank database under accession numbers of PV620908 for *Lactococcus lactis* CRAC, PV620909 for *Levilactobacillus brevis* CRAI, PV620910 for *Levilactobacillus brevis* CRAR, PV620911 for *Levilactobacillus brevis* DRBA2, PV620912 for *Levilactobacillus brevis* DVBA2, PV620913 for *Lactococcus lactis* IBA, PV620914 for *Lactococcus lactis* IBB, PV620915 for *Lactococcus lactis* PBMC, PV620916 for *Lactococcus lactis* PBMD, PV620917 for *Lactiplantibacillus plantarum* PRA, PV620918 for *Lactiplantibacillus plantarum* DRBA1, PV620919 for *Lactiplantibacillus plantarum* DVBA1, and PV620920 for *Levilactobacillus brevis* DVB22, respectively. The genome sequence of *L. brevis* CRAI was submitted to NCBI GenBank under accession number SAMN48404542. The strain *L. brevis* CRAI has been deposited in the DSMZ collection under the deposition number DSM 35345.

### 2.11. Data and Statistical Analysis

Data referred to HPLC quantification are presented as means of two independent experiments ± standard deviation (SD). Antimicrobial and anti-inflammatory activities were expressed as mean ± SD of two independent experiments, and one-way ANOVA followed by Bonferroni correction was used for multiple comparisons. Differences were deemed significant for *p* < 0.05. All statistical analyses were performed using GraphPad Prism version 9.5.1 for Windows.

## 3. Results

### 3.1. Taxonomic Characterisation of LAB from Tomato and gadB Gene Detection

Starting from six tomato varieties, 29 colonies showing different morphologies were obtained on MRS plates, under selective anaerobic conditions. Among those, 23 colonies showed rod-shaped cells at microscopic observation and were further analysed; 16 isolates were found to be LAB. Such isolates were taxonomically identified at the species level by sequencing the 16S rRNA gene and aligning the sequence with the BLAST database. We found five *Levilactobacillus brevis* (DRBA2, DVBA2, DVBA22, CRAI, CRAR), three *Lactiplantibacillus plantarum* (DRBA1, DVBA1, PRA), and five *Lactococcus lactis* (CRAC, IBA, IBB, PBMD, PBMC) strains (Table 1). To identify which isolates were potential GABA producers, LAB strains were screened for the presence of the *gadB* gene by PCR amplification. The *gadB* gene was detected in all thirteen LAB strains (Table 1).

### 3.2. HPLC Quantification of GABA and Selection of L. brevis CRAI Strain

Since all the LAB strains bear the *gadB* gene, they were all tested for their capacity to produce GABA. According to the information reported in the literature [30], fermentations were carried out by adding 236.53 mM (4%) MSG to the medium to maximise GABA production, and GABA was quantified by HPLC analysis. All tested isolates produced quantifiable amounts of GABA, although variable among strains, ranging from 0.73 to 179.15 mM. Within *L. brevis* strains, the highest amount of GABA was produced by CRAI (179.15 mM) and CRAR (165.24 mM) isolates (Table 2). On the contrary, a lower quantity of GABA was produced by the strains belonging to *L. plantarum* and *L. lactis* species. The strains *L. brevis* CRAI and CRAR exhibited a conversion yield of 75.75% and 69.88%, respectively (Table 2). These data evidenced that *L. brevis* CRAI is the best GABA producer, displaying the highest conversion rate of MSG to GABA.

### 3.3. GABA Production When L. brevis CRAI Was Cultivated with Different MSG Concentrations

The selected strain, *Levilactobacillus brevis* CRAI, was cultured under optimised conditions [30], and different concentrations of MSG, ranging from 0 to 4% (0–236.5 mM), were added to MRS medium. GABA production resulted in being dependent on MSG concentration: *L. brevis* CRAI produced 6.94 ± 0.48 mM of GABA in the absence of MSG (0%) and increased productivity to 179.15 ± 15.14 mM when MSG was added at 236.5 mM (4%) (Table 3).

### 3.4. L. brevis CRAI Genome Features

**Figure 1:**
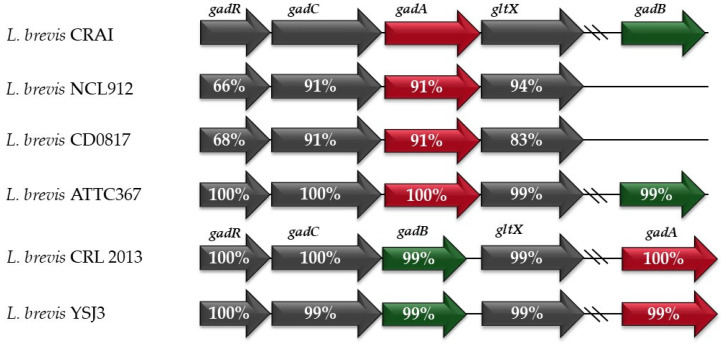
Gene organisation of GAD operon in *Levilactobacillus brevis* CRAI and other *L. brevis* strains. Percentages of identity between each aminoacidic sequence and *L. brevis* CRAI one are reported inside the arrows; *gadR*, transcriptional regulator; *gadC*, glutamate/gamma-aminobutyric acid antiporter gene; *gadA* (red color), glutamate decarboxylase A gene; *gltX*, glutamate-tRNA ligase gene; *gadB* (green color), glutamate decarboxylase B gene. Arrows indicate gene orientations.

The genome sequence of the *L. brevis* CRAI strain was deposited in GenBank (accession number SAMN48404542). The genome comprises a 2,550,312 bp circular chromosome with an average GC content of 45.9%. The annotation showed the presence of two genes coding for glutamate decarboxylase, corresponding to two isoforms of GAD known as *gadA* and *gadB* genes [38]. The two genes showed a sequence identity of 64% and are located in two distinct loci on the chromosome. *gadA* is part of a gene cluster where the transcriptional regulator *gadR* is positioned at the upstream end, followed by the glutamate/gamma-aminobutyric acid antiporter *gadC*, the glutamate decarboxylases *gadA*, and the *gadX* gene, encoding for glutamate-tRNA ligase. The *gadB* gene is not included in the gene cluster and is located 1.7 Mbp apart from *gadA* (Figure 1). Comparing the amino acidic sequences of the proteins involved in GABA production by *L. brevis* CRAI with other strains of the same species reported in the literature, a very high identity was found (Figure 1). In particular, *L. brevis* CRAI shared the same sequence of gadA with *L. brevis* ATTC367 and *L. brevis* CRL2013, although these two latter isolates showed a different locus organisation. In *L. brevis* CRAI, *L. brevis* NCL912, *L. brevis* ATTC367, and *L. brevis* CD0817, the *gadA* gene is located within the *gad* operon, while in *L. brevis* CRL2013 and YSJ3, it is *gad*B that is found inside the operon [10,38,44]. The gadB protein sequence of these strains was 99% identical to that of *L. brevis* CRAI. Another strain showing a high percentage of amino acid sequence identity to the proteins of *L. brevis* CRAI is *L. brevis* YSJ3, whereas gad proteins of *L. brevis* NCL912 and CD0817 show lower sequence similarity; for instance, the gadA protein in both NCL912 and CD0817 shares only 91% identity with that of *L. brevis* CRAI.

Notably, genome annotation pointed to the presence of genes that can be related to beneficial properties: the *bsh* gene encodes for choloylglycine hydrolase, a bile salt hydrolase (BSH) that catalyses the hydrolysis of the amide bond and its activity contributes to cholesterol level reduction [45]. Additionally, the presence of genes involved in riboflavin synthesis was detected: specifically, the *rib* operon, containing the genes for riboflavin synthesis *rib*G, *rib*B, *rib*A, *rib*E, and *rib*H, was identified, with a regulatory element (RFN) at the end of the operon [46]. The strain’s safety profile was analysed using various tools, including AMRFinderPlus, ResFinder, and CARD. The first two tools did not identify any genes associated with antibiotic resistance or virulence; CARD detected the *vanT* and *nimA* genes. The *vanT* gene encodes for an alanine racemase enzyme, while *nimA* encodes a pyridoxamine 5′-phosphate oxidase superfamily protein involved in vitamin B6 metabolism. However, these genes are not associated with antimicrobial resistance.

The sequencing of *L. brevis* CRAI DNA also revealed the presence of two natural plasmid-related sequences. As revealed by RAST analysis, the plasmids bear genes involved in potassium homeostasis and bile hydrolysis, while virulence factor or antibiotic-resistance-related genes are absent.

### 3.5. Transcriptional Analysis of gadB and gadA Genes in L. brevis CRAI

To investigate the impact of the addition of MSG to the medium on the expression level of the *gadA* and *gadB* genes, we conducted RT-PCR experiments on mRNA extracted from *L. brevis* CRAI cells cultured in optimised conditions (10^7^ CFU/mL inoculum, pH adjusted to 5, 32 °C, 48 h of incubation) in the presence of 236.53 mM (4%) MSG. *gadA* and *gadB* gene expression was compared to the basal condition (MSG concentration: 0). The data indicated that the supplementation of MRS medium with 4% MSG led to a 52.52-fold increase in *gadA* mRNA and a 4.44-fold increase in *gadB* mRNA.

### 3.6. Antimicrobial Activity of L. brevis CRAI

Given the growing interest in beneficial bacterial strains exhibiting multiple functionalities, including the capability to modulate the gut–brain axis via bioactive compounds, *L. brevis* CRAI was also investigated for its probiotic potential. Among major health-promoting activities of probiotics, the ability to counteract gastrointestinal infections was assessed [45]. Therefore, enterotoxigenic *E. coli*, *S. choleraesuis*, and *Y. enterocolitica* were chosen as representative pathogen strains responsible for intestinal infections. We tested the effectiveness of the cell-free supernatant of *L. brevis* CRAI in inhibiting the planktonic growth of these gut pathogens, and it was able to almost abolish the growth of intestinal pathogens: inhibition percentages were 97.24 ± 1.53% and 97.53 ± 0.74% for *E. coli* ETEC and *S. choleraesuis*, and 99.12 ± 0.67% for *Y. enterocolitica* (Table 4). The antagonistic activity exhibited by *L. brevis* CRAI viable cells was also evaluated by overlay assay. We found that CRAI viable cells possess a remarkable inhibitory activity with an inhibition zone above 16 mm against *E. coli* ETEC and *S. choleraesius*, and above 20 mm against *Y. enterocolitica* (Table 4).

### 3.7. Anti-Inflammatory Activity of L. brevis CRAI

**Figure 2:**
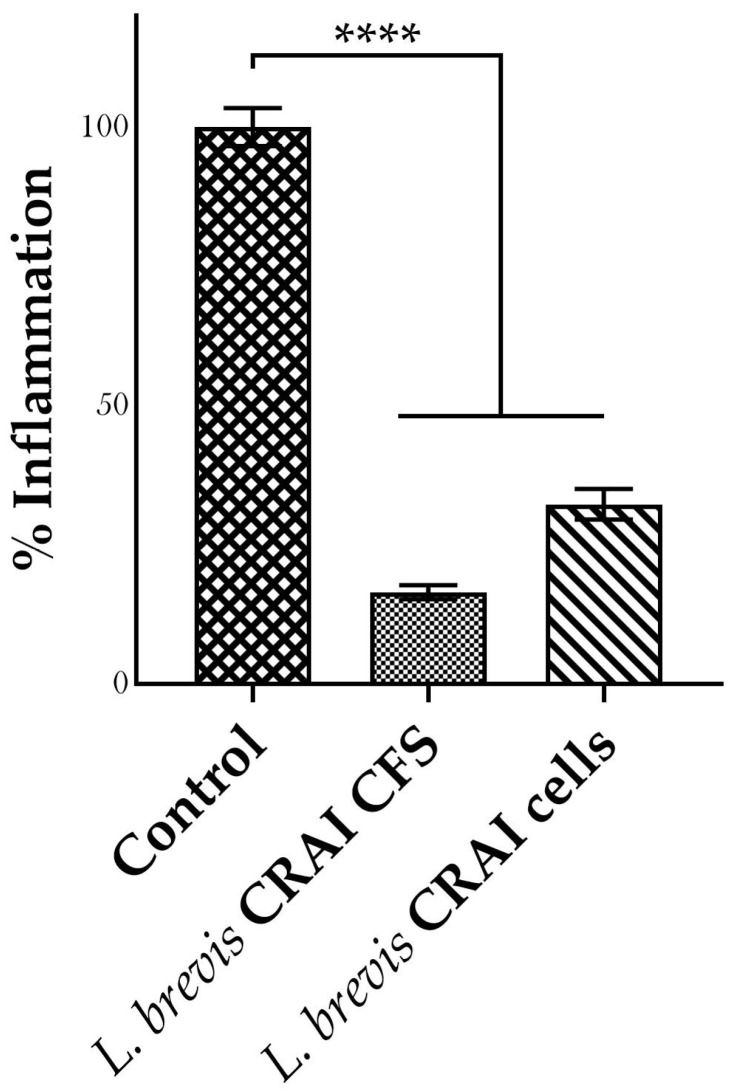
Anti-inflammatory activity of CFS and viable cells of *L. brevis* CRAI. RAW264.7 cells stimulated with LPS were treated with *L. brevis* CRAI CFS or cells. NO levels are expressed as % of inflammation, with respect to control (100%). **** *p* < 0.0001.

An imbalanced gut microbiota, or dysbiosis, is often associated with inflammation, which can also be one of its underlying causes. As recent strategies focus on restoring gut balance through probiotics administration [46], we sought to investigate the anti-inflammatory activity of *L. brevis* CRAI. The RAW264.7 cell line is widely used to study immune responses, particularly in the context of inflammation. Lipopolysaccharide (LPS) is known to activate macrophages, triggering the release of various pro-inflammatory cytokines and mediators of inflammation such as nitric oxide (NO). To assess nitric oxide (NO) production, which is indicative of the inflammatory response in macrophages, we conducted the Griess reaction assay, in which RAW 246.7 cells were stimulated with LPS, and the NO level was assessed in the presence or absence of *L. brevis* CRAI. As illustrated in Figure 2, treatment of LPS-induced macrophages with either *L. brevis* CRAI cell-free supernatant or bacterial cells resulted in a significant reduction of inflammation, to 16.50 ± 1.24% and 32.33 ± 2.69%, respectively, compared to the control (100%) (Figure 2).

### 3.8. Ability of L. brevis CRAI to Adhere to the Intestinal Epithelium

We then evaluated the capability of *L. brevis* CRAI to adhere to the Caco-2 cell line, taken as an in vitro model of intestinal epithelium. We applied a quantitative method based on adherent cell counting. *L. brevis* CRAI exhibited the capacity to adhere to Caco-2 cells, and the ratio of viable adherent *L. brevis* cells to Caco-2 cells was 3.06 ± 0.71:1.

## 4. Discussion

GABA is recognised as one of the most important inhibitory neurotransmitters, playing a crucial role in various physiological functions such as the regulation of the sleep–wake cycle, motor activity, and vascular tone [2]. The metabolic role of GABA involves supplying energy to the brain while enhancing its resilience against oxygen deprivation and various stressors [2]. GABA is used in food and drugs exhibiting antihypertensive, analgesic, and antidepressant properties [3,4]. It is well established that GABA is a metabolite produced by the lactobacilli residing in the intestinal tract, and its production is associated with beneficial effects on the host [4]. The objective of this study is to identify a lactic acid bacterium that demonstrates efficient GABA production and, at the same time, valuable probiotic properties, in the perspective of selecting a beneficial strain with multiple functionalities. To achieve this, six different tomato varieties were used to isolate LAB strains, which were subsequently taxonomically identified by 16S rRNA gene sequencing. We identified 13 different strains belonging to the species *Levilactobacillus brevis*, *Lactiplantibacillus plantarum*, and *Lactococcus lactis*. LAB strains capable of synthesising GABA harbour within their genome the *gad* gene, which is essential for this biochemical pathway [10]. Interestingly, LAB strains can possess one or two homologous GAD enzymes, encoded by *gadA* and *gadB* genes [18]. In this study, we first selected strains able to produce GABA based on the PCR detection of the *gadB* gene, which is the most prevalent and conserved among LAB [47]. PCR analyses confirmed the presence of the *gadB* gene in all thirteen isolates, suggesting them as putative GABA producers.

To assess the ability of LAB isolates to convert glutamate into GABA, we subsequently set up fermentations in optimised conditions. It is known that GABA production is influenced by multiple factors: Wu CH et al. (2018) [19] demonstrated how *L. brevis* RK03’s efficiency to produce GABA was affected by the fermentation parameters, such as temperature, pH, and glutamate concentration. The glutamate-dependent mechanism is associated with increased acid resistance observed in various bacterial strains (21); indeed, adjustment of the pH medium modulates the production of GABA. Moreover, GABA production is dependent on MSG availability, and high levels of MSG (6% to 15%) suppressed GABA production [48]. To identify the strains with the highest capacity of converting glutamate to GABA, we cultured LAB strains in optimised conditions according to data in the literature [18,30,31,32] and quantified GABA by HPLC. All tested LAB strains were able to produce GABA, although with variable efficiency. We selected the best GABA producer, i.e., the *L. brevis* CRAI strain, which exhibited a conversion yield of 75.75% and a GABA production of 179.15 mM starting from an initial concentration of 236.53 mM (4%) of MSG. Recent studies confirm that among LAB, *L. brevis* is the species that demonstrates the highest efficiency in GABA production [20,49,50]. Li H. 2010 [48] reported that GABA production can be enhanced by optimising the culture conditions, for example, the initial glutamate concentration had significant effects on the cell growth of *L. brevis* NCL912 and on its conversion into the neurotransmitter. Consequently, we tested different concentrations of MSG and measured the output (GABA). The results showed that GABA production was the highest in the presence of MSG 236.53 mM (4%). Since multiple factors influence GABA production levels, it is difficult to compare GABA productivity achieved in different studies, where different protocols were adopted. However, culture conditions used in the present study were similar to those employed by Villegas J et al. (2016) with *L. brevis* CRL1942, where a production level of 255 mM of GABA with 4.5% MSG was reported. Such productivity is in line with our results. Notably, probiotic activities were not characterised in the *L. brevis* CRL1942 strain [51].

The genome sequence of *L. brevis* CRAI was analysed to predict its functional characteristics and to determine the arrangement of the genes involved in GABA production. The genome annotation revealed that *L. brevis* CRAI contains both *gadA* and *gadB* genes. The *gadA* gene is located in the *gad* operon, whereas *gadB* is located separately from the operon (Figure 1). This genetic organisation was also found in *L. brevis* ATCC367, where *gadA* is in the operon, far from *gadB* [52]. High levels of aminoacidic sequence identity were found in *L. brevis* isolates for both gadA and gadB proteins, despite different locus organisation, indicating a high degree of sequence conservation. Furthermore, genomic analysis revealed important putative probiotic and functional genes, such as genes associated with bile salt hydrolase and the riboflavin synthetic pathway. The gene encoding for choloylglycine hydrolase enzyme (BSH) is responsible for bile salt hydrolysis. It is involved in the bacterial tolerance to bile salt as it facilitates the utilisation of amino acids as carbon and nitrogen sources, driving the bile detoxification with hypocholesterolaemic effects [53]. It has been proposed that BSH from gut microbiota and beneficial microbes influences the crosstalk between gut microbiota and host health, by modulating the bile acid landscape [54]. Many LAB strains produce a range of metabolites, including B-vitamins, such as riboflavin and folate, and low-calorie sugars, such as exopolysaccharides, which could be employed in the food and dairy industry [55]. The enzymes required to catalyse the biosynthesis of riboflavin from guanosine triphosphate and ribulose-5-phosphate are encoded by five genes: *rib*G, *rib*B, *rib*A, *rib*E, and *rib*H, as described by Perkins et al. 1999 [56]. The gene order within the operon differs from the order of enzymatic reactions that lead to riboflavin biosynthesis. In the *L. brevis* CRAI genome, this operon was detected together with the regulatory region RFN that transcribed the genes. The potential of a probiotic strain to produce riboflavin is of great interest, since it is an essential metabolite that humans cannot synthesise. In situ production of riboflavin in the human gut by beneficial bacteria can directly benefit the host, besides helping to stabilise the bifidobacteria commensal species [57]. The ability to withstand bile salt stress and to synthesise vitamin B represents an added value that evidences the probiotic potential of the strain. Furthermore, different tools were used and confirm the safety profile of *L. brevis* CRAI. CARD’s Resistance Gene Identifier (RGI) software detected two putative antimicrobial resistance (AMR) genes, *vanT* and *nimA*, both displaying low sequence identities (32% and 48%, respectively). These genes have also been identified in *L. brevis* CRL 2013 strain, in which the *vanT* gene, a member of the racemase family, was reported to be essential for the growth of LAB and was not linked to the *vanG* cluster associated with vancomycin resistance. The authors also identified the *nimA* gene that encodes an enzyme involved in vitamin B6 metabolism [58]. The DNA sequencing revealed the presence of two natural-plasmid-related sequences, which were found in other *L. brevis* strains [59,60]. Gene annotation showed that the two plasmids bear genes involved in potassium homeostasis and in bile hydrolysis, while virulence genes and antibiotic resistance genes were not found. We can conclude that *L. brevis* CRAI could be considered safe from a genetic point of view, since no virulence factors or antibiotic-resistant genes were found.

Genomic analysis of *L. brevis* CRAI identified two genes, *gadA* and *gadB*, involved in GABA production. To explore whether the observed differences in GABA production are linked to variations in gene expression, we examined the expression levels of these genes in response to two different MSG concentrations, 0 and 236.53 mM (4%) added to the medium. The expression of the mRNA of the *gadA* and *gadB* genes increased 52.52-fold and 4.44-fold, respectively, in the presence of 4% MSG (236.53 mM), compared to the conditions without MSG. These results suggest that both genes are induced by the presence of glutamate. Notably, the *gadA* gene shows a stronger and more pronounced induction, likely due to its location within an operon that contains a regulatory region upstream, i.e, *gadR*. It has been reported that, in *L. lactis* isolates, the transcriptional regulator gadR induces the expression of downstream genes in response to the presence of glutamate in the growth medium. In *L. brevis* ATCC367, disrupting the *gadR* gene impaired GABA production by reducing the expression of the *gadC* and *gadA* genes [58]. Therefore, we can speculate that the transcriptional regulation sequence *gadR* upstream of the operon may be activated by the presence of glutamate, highly inducing *gadA* transcription.

Lactic acid bacteria, thanks to their remarkable functional properties, represent promising probiotic candidates. The characterisation of probiotic properties includes antagonistic activity against pathogens, anti-inflammatory potential, and adhesion to epithelial cells [61]. In this study, we assessed the inhibitory effects of *L. brevis* CRAI against *E. coli* ETEC, *S. choleraesuis*, and *Y. enterocolitica*. First, we demonstrated the ability of *L. brevis* CRAI cell-free supernatants to significantly limit enteropathogens’ planktonic growth. Then we determined the ability of the *L. brevis* CRAI viable cells to limit pathogens viability. Both fractions of *L. brevis* exhibited antibacterial activities against enteropathogens, with greater effects against *Y. enterocolitica.* This antimicrobial activity is likely attributed to the production of organic acids, hydrogen peroxide, bacteriocins, and other molecules, as reported in studies on other *L. brevis* strains [62].

Among the probiotic properties of functional strains, anti-inflammatory activity is of great interest [63]. Upon stimulation, macrophages, the key players of inflammation, recognise foreign threats by specific receptors and initiate an inflammatory response. However, the overproduction of proinflammatory mediators, such as NO, causes an excessive macrophage activation that can lead to a chronic condition, including inflammatory bowel disease and arthritis [64]. Therefore, the present study aims to determine the effects of *L. brevis* CRAI on the inflammatory response in RAW264.7 macrophages. *L. brevis* CRAI significantly reduced the inflammation triggered by LPS; in fact, the percentage of inflammation was reduced to 16% and 32% when macrophages were treated with CFS and cells, respectively. Interestingly, it was reported that *L. brevis* KU15147 inhibits nitric oxide and prostaglandin E_2_ levels by decreasing the activation of inducible nitric oxide synthase and cyclooxygenase-2 without cell cytotoxicity [65]. Our findings demonstrated the inflammatory inhibition exerted by *L. brevis* CRAI.

The colonisation of the intestinal wall is considered a desirable property of probiotic bacteria [66]. Liu et al. demonstrate that different strains of *L. brevis* showed adhesion capacity to Caco-2 cells ranging from 1.98 to 8.53% [50]. Similarly, our findings indicate that *L. brevis* CRAI exhibits the capacity to adhere to the Caco-2 cell line, at a ratio of three bacteria per cell.

## 5. Conclusions

In conclusion, LAB strains were isolated from organic tomatoes, screened for the presence of the *gadB* gene, and then tested for GABA production. We selected *L. brevis* CRAI strain as the best GABA producer because it converted most of the glutamate added to the medium into GABA. Furthermore, we demonstrated that GABA production depends on the presence of glutamate in the medium. This finding is further supported by the analysis of the *gadA* and *gadB* gene expression; both genes, especially *gadA*, were induced when the glutamate concentration in the medium was present at 4%. The *L. brevis* CRAI genome analysis evidenced the presence of the GAD operon and important genes that confer probiotic features, such as the bile salt hydrolase gene and riboflavin pathway genes, and excluded the presence of genes related to antimicrobial resistance and virulence factors. This strain showed antimicrobial and anti-inflammatory activity and the ability to adhere to Caco-2 cells. This strain satisfies more than one of the characteristics that support its potential as a promising probiotic candidate. Notably, the beneficial activity of *L. brevis* CRAI fulfilled the growing interest in functional probiotics that can modulate the gut–brain axis via bioactive compounds.

## Figures and Tables

**Table 1:** Taxonomic characterisation of LAB isolates by 16S rRNA gene sequencing and detection of *gadB* gene.

Tomato Variety	Species	Strain	*gadB* Gene
Grape Red	*Levilactobacillus brevis*	DRBA2	+
	*Lactiplantibacillus plantarum*	DRBA1	+
Grape Green	*Lactiplantibacillus plantarum*	DVBA1	+
	*Levilactobacillus brevis*	DVBA2	+
	*Levilactobacillus brevis*	DVBA22	+
Cherry Red	*Levilactobacillus brevis*	CRAI	+
	*Levilactobacillus brevis*	CRAR	+
	*Lactococcus lactis*	CRAC	+
Plum	*Lactococcus lactis*	IBA	+
	*Lactococcus lactis*	IBB	+
Beefsteak	*Lactococcus lactis*	PBMD	+
	*Lactococcus lactis*	PBMC	+
Roma	*Lactiplantibacillus plantarum*	PRA	+

**Table 2:** GABA quantification by HPLC and conversion yield. Fermentations were carried out in the presence of MSG 236.53 mM (4%).

Species	Strain	GABA mM	Conversion Yield %
*Levilactobacillus brevis*	CRAI	179.15	75.75
	CRAR	165.24	69.88
	DVBA2	7.10	3.00
	DVBA22	4.71	1.99
	DRBA2	0.73	0.31
*Lactiplantibacillus plantarum*	DRBA1	2.91	1.23
	DVBA1	2.25	0.95
	PRA	3.32	1.40
*Lactococcus lactis*	CRAC	8.24	3.49
	IBA	36.21	15.31
	IBB	1.99	0.84
	PBMD	0.97	0.41
	PBMC	34.59	14.63

**Table 3:** GABA production by *L. brevis* CRAI in the presence of different MSG concentrations. Data are expressed as the mean of two independent experiments ±SD.

Strain	MSG mM (%)	GABA mM
*Levilactobacillus brevis* CRAI	0	6.94 ± 0.48
	59.1 (1%)	48.32 ± 5.98
	118.2 (2%)	77.66 ± 16.46
	177.3 (3%)	116.29 ± 13.96
	236.5 (4%)	179.15 ± 15.14

**Table 4:** Inhibition of pathogens by *L. brevis* CRAI CFS and viable cells. The enteropathogens tested were *E. coli* ETEC, *S. choleraesuis* serovar Typhimurium, *Y. enterocolitica*. The % of inhibition was assessed by comparing the turbidity (OD600) of the sample treated with CFS with the untreated control (0%). The antagonistic activity showed by the strain’s viable cells was expressed as +, radius between 14–18 mm; ++, radius > 18 mm.

*L. brevis* CRAI	*E. coli* ETEC	*S. choleraesius* Typhimurium	*Y. enterocolitica*
CFS (%)	97.24 ± 1.53	97.53 ± 0.74	99.12 ± 0.67
Viable cells (mm)	+	+	++

## Data Availability

The data presented in this study are openly available in ASM Acta at https://doi.org/10.6092/unibo/amsacta/8349. 16S sequences and the *L. brevis* CRAI genome are available in NCBI GeneBank.
